# Failures of cognitive control or attention? The case of stop-signal deficits in schizophrenia

**DOI:** 10.3758/s13414-017-1287-8

**Published:** 2017-02-09

**Authors:** Dora Matzke, Matthew Hughes, Johanna C. Badcock, Patricia Michie, Andrew Heathcote

**Affiliations:** 1 Department of Psychology, University of Amsterdam, Postbus 15906, 1001 NK Amsterdam, The Netherlands; 2 Brain and Psychological Sciences Centre, Swinburne University of Technology, Hawthorn, Australia; 3 Centre for Clinical Research in Neuropsychiatry, School of Psychiatry and Clinical Neurosciences, The University of Western Australia, Crawley, Australia; 4 School of Psychology and Centre for Brain and Mental Health Research, University of Newcastle, Callaghan, Australia; 5 School of Medicine, University of Tasmania, Hobart, Australia

**Keywords:** Stop-signal paradigm, Inhibition deficits, Attention deficits, Trigger failure, Schizophrenia

## Abstract

We used Bayesian cognitive modelling to identify the underlying causes of apparent inhibitory deficits in the stop-signal paradigm. The analysis was applied to stop-signal data reported by Badcock et al. (Psychological Medicine 32: 87-297, 2002) and Hughes et al. (Biological Psychology 89: 220-231, 2012), where schizophrenia patients and control participants made rapid choice responses, but on some trials were signalled to stop their ongoing response. Previous research has assumed an inhibitory deficit in schizophrenia, because estimates of the mean time taken to react to the stop signal are longer in patients than controls. We showed that these longer estimates are partly due to failing to react to the stop signal (“trigger failures”) and partly due to a slower initiation of inhibition, implicating a failure of attention rather than a deficit in the inhibitory process itself. Correlations between the probability of trigger failures and event-related potentials reported by Hughes et al. are interpreted as supporting the attentional account of inhibitory deficits. Our results, and those of Matzke et al. (2016), who report that controls also display a substantial although lower trigger-failure rate, indicate that attentional factors need to be taken into account when interpreting results from the stop-signal paradigm.

The capacity to inhibit action as required by changes in the environment or internal states is essential for ensuring coherent action and enables contextually relevant and goal-directed behaviour. Response inhibition is typically assessed using the stop-signal task (Fig. 1), where action execution in response to a choice “go” stimulus is supposed to be inhibited on a small proportion of trials in response to a subsequent stop signal (Verbruggen & Logan, 2008). Although the duration of go response processes can be measured directly using go reaction time (RT) on trials without stop signal, the duration of the stop process is not directly observable, and so has to be inferred.

**Fig. 1:**
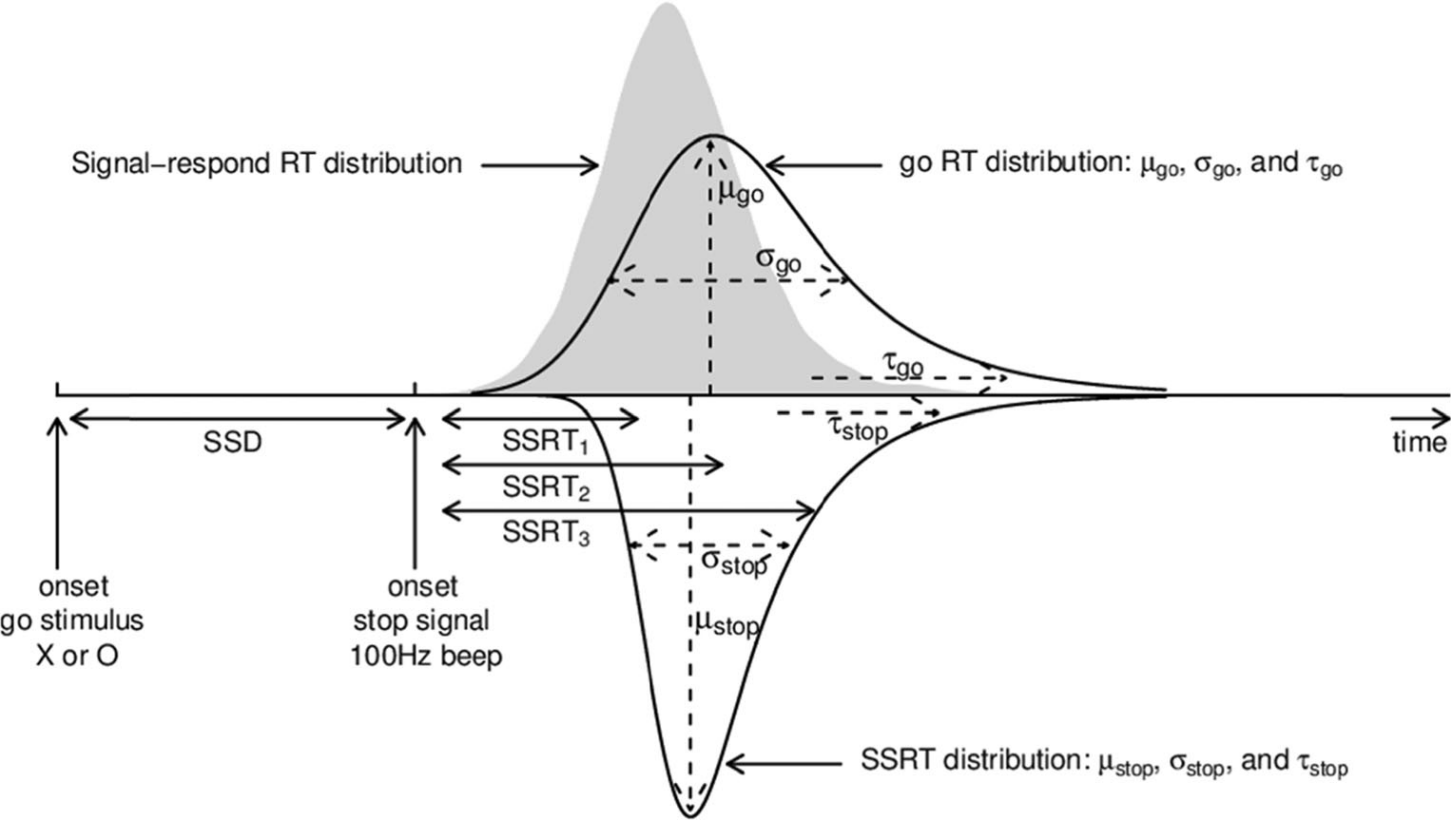
Stop-signal paradigm and the corresponding horse-race model. In the stop-signal paradigm, participants perform a choice RT task (i.e., the go task), such as responding to the shape of the go stimulus (e.g., press a left key for “X” and a right key for “O”). Occasionally, the go stimulus is followed by a stop signal (e.g., a 1000-Hz auditory tone) after a variable stop-signal-delay (SSD), instructing participants to withhold their response. Performance in the stop-signal paradigm is modelled as a horse-race between two independent processes: go process and stop process (Logan & Cowan, 1984). The finishing times of the go and stop processes are assumed to be random variables that follow an ex-Gaussian distribution, with parameters μ, σ, and τ. On a given trial, if the go RT is slower than SSD + SSRT, the go response is inhibited; if the go RT is faster than SSD + SSRT, the go response cannot be inhibited and results in a signal-respond RT (i.e., grey distribution)

Stop-signal performance has been modelled as a race between independent go and stop processes that are triggered by go and stop signals, respectively. On stop-success trials, the stop process accumulates activation sufficiently quickly to achieve threshold and inhibit responding before the go process can reach its threshold; hence the stop process wins the race. On stop-failure trials, the go process reaches threshold first and wins the race. The outcome of the race is determined by the speeds of the go and stop processes and by the delay between the go and stop signal (stop-signal delay [SSD]; Logan & Cowan, 1984). A summary measure of inhibitory ability in the form of the mean time for the stop process to reach threshold (i.e., stop-signal RT or SSRT) can be estimated nonparametrically by assuming that go RT distributions for trials with and without stop signal are the same (e.g., Band et al., 2003).

Stop-signal tasks have been used extensively in studies of schizophrenia patients and their relatives (Badcock et al., 2002; Ross et al., 2008). General slowing in mean RT for simple manual choice tasks is a pervasive symptom of schizophrenia (Heathcote et al., 2015; Kieffaber et al., 2006; Schatz 1998), and prolonged SSRTs are commonly reported (Bellgrove et al., 2006; Enticott et al., 2008; Hughes et al., 2012; Thakkar et al., 2011, 2015). Prolonged SSRT in patients has been regarded as indicative of impaired inhibitory processing (Lipszyc et al., 2010).

However, successful stopping requires not only short SSRTs but also the capacity to trigger inhibitory processes. Trigger failures pose long-known challenges to the interpretation of stop-signal data, because apparent group differences in inhibition performance may result from differences in SSRT, but they might just as well reflect differences in the probability of triggering the stop process (Logan, 1994). At least one study has proposed that the major deficit in schizophrenia may lie in trigger failures. Badcock et al.’s (2002) proposal was based on a nonparametric measure derived from inhibition functions–plots of the probability of stop failures for a range of SSDs. However, Band et al. (2003) showed that even appropriately transformed inhibition functions are unable to discriminate between trigger failures and differences in go RT and SSRT variabilty.

Recently, Matzke et al. (2016) developed a parametric (model-based) Bayesian approach that addresses this problem. They showed that ignoring trigger failures leads to dramatic overestimation of SSRTs and that trigger failures occurred on approximately 10% of trials for both Badcock et al.’s (2002) and Hughes et al.’s (2012) healthy controls. As a result, a recent methodological review of the stop-signal paradigm (Matzke, Verbruggen, & Logan, in press) stressed the importance of accounting for failures to trigger the stop process.

Here we apply Matzke et al.’s (2016) Bayesian approach to data reported for schizophrenia patients in Badcock et al. (2002) and Hughes et al. (2012) to determine to what degree increased trigger failures can explain deficits in patients’ stop-signal performance. As trigger failures indicate an attentional deficit (e.g., a failure in encoding the stop signal) rather than an inhibitory deficit, our analysis could potentially change the traditional interpretation of prolonged SSRTs in patients from a dysfunction of cognitive control (Barch, 2005) to a dysfunction of attention (Braff, 1993). This interpretation also would serve to validate and generalize Matzke et al.’s argument that attentional factors need to be taken into account in broader applications of the stop-signal paradigm.

## Bayesian Cognitive Modelling

Matzke et al.’s (2016) Bayesian hierarchical approach simultaneously estimates the probability of trigger failures and the full distribution (as opposed to only the mean) of go RTs and SSRTs. Hierarchical modelling provides inference on both the participant and the population level, and can provide more accurate and less variable estimates than individual estimation (Farrell & Ludwig, 2008). As shown in Fig. 1, the model is based on the complete horse-race model that treats both go RTs and SSRTs as random variables (Logan & Cowan, 1984). On a given trial, if the go RT is slower than SSD + SSRT, the go response is inhibited; if the go RT is faster than SSD + SSRT, the go response cannot be inhibited and results in a signal-respond RT (i.e., grey distribution).

The model assumes that go RTs and SSRTs follow an ex-Gaussian distribution (see also Matzke, Dolan et al., 2013), which is the sum of a normal distribution with mean μ and standard deviation σ, and an exponential distribution with mean τ (i.e., the tail of the distribution; see Fig. 1). The mean of the ex-Gaussian distribution is the sum of μ and τ; hence, mean go RT is given by μ_go_+τ_go_ and mean SSRT by μ_stop_+τ_stop_. In addition to the ex-Gaussian go and stop parameters, Matzke et al.’s (2016) extension of the model also estimates a parameter, P_TF_, that quantifies the probability that participants’ fail to trigger the stop process altogether.

Matzke and Wagenmakers (2009) discussed how the ex-Gaussian parameters could be interpreted in terms of the widely adopted view that RT can be explained in terms of “accumulate-to-threshold” processing, and in particular in terms of the cognitive processes assumed by the diffusion decision model (Ratcliff & McKoon, 2008). In this model, after a stimulus had been encoded it provides evidence that causes a change of activation in an accumulation process, with a faster rate of accumulation for stimuli that provide stronger evidence. When activation reaches a threshold, response production is triggered. The threshold determines the amount of activation required to make a response. Participants can set the threshold strategically in order to control the trade-off between speed and accuracy (e.g., a higher threshold causes slowing, but reduces errors because responses are based on more evidence). RT is the sum of encoding, accumulation, and response production times.

Through a series of data simulations based on varying diffusion model parameters, Matzke and Wagenmakers (2009) demonstrated that slowing due to τ is associated only with characteristics of the accumulation processes (i.e., a slower rate of increase in activation or higher threshold). Slowing due to μ is associated with higher thresholds but not with rates, and can also reflect deficits outside the accumulation process, such as slower initiation of these processes due to stimulus-processing deficits, or slower response production.

Based on these considerations, separate estimates of μ and τ provide greater insights than standard measures of mean SSRT into the causes of patient deficits. Specifically, an increase in SSRT due to μ_stop_ is likely caused by deficits in the processing of the stop signal, which slows the triggering of the stop process, and like trigger failures would be indicative of attentional deficits. In contrast, an increase in SSRT due to τ_stop_ is likely caused by a reduced rate in the stop process, and hence would be indicative of inhibitory deficits^1^.

In order to address the divergent validity of these parameters, we also examine the causes of slowing in patients’ go RTs. The same pattern of effects on go and stop μ and τ estimates would suggest similar and perhaps common underlying causes, whereas a contrasting pattern would suggest different causes of any slowing in stop and go processes. As the data sets we examine used very easy choice tasks with high accuracy, go threshold differences, which are usually associated with strategic attempts to control errors, are unlikely. The tasks also relied on simple button press responses, which are not associated with response production deficits in schizophrenia (Heathcote et al., 2015). Hence, any patient deficits in μ_go_ are likely caused by stimulus encoding delays.

## Methods

### Data sets

Detailed experimental methods are provided in Badcock et al. (2002) and Hughes et al. (2012); we highlight aspects relevant to our analysis. Both studies used go tasks requiring fast, accurate responses to equi-probable “O” and “X” stimuli. Stop signals were 1000-Hz tones presented for 100 ms on 25% of the trials. Badcock et al. used a range of six 100-ms spaced SSDs relative to mean go RT in the preceding block. Hughes et al. set SSDs adaptively: after stop failures SSD decreased by 50 ms; after stop successes it increased by 50 ms.

For Badcock et al. (2002) we analysed data from 17 schizophrenia patients and 30 controls (removing four patients with go error rates greater than 10% and two patients and one control with unusual left-skewed go RT distributions).^2^ We used only correct RTs and removed RTs faster than 250 ms. We also removed go RTs slower or faster than mean RT ±3 standard deviations, resulting in an average data loss of 5% of the trials in the schizophrenia and 4% in the control group. For Hughes et al. (2012), we used all 10 and 13 participants in the schizophrenia and control groups, respectively. We used only correct RTs and removed RTs faster than 200 ms, resulting in an average data loss of 7% of the trials in the schizophrenia and 3% in the control group.

### Bayesian analysis

**Fig. 2:**
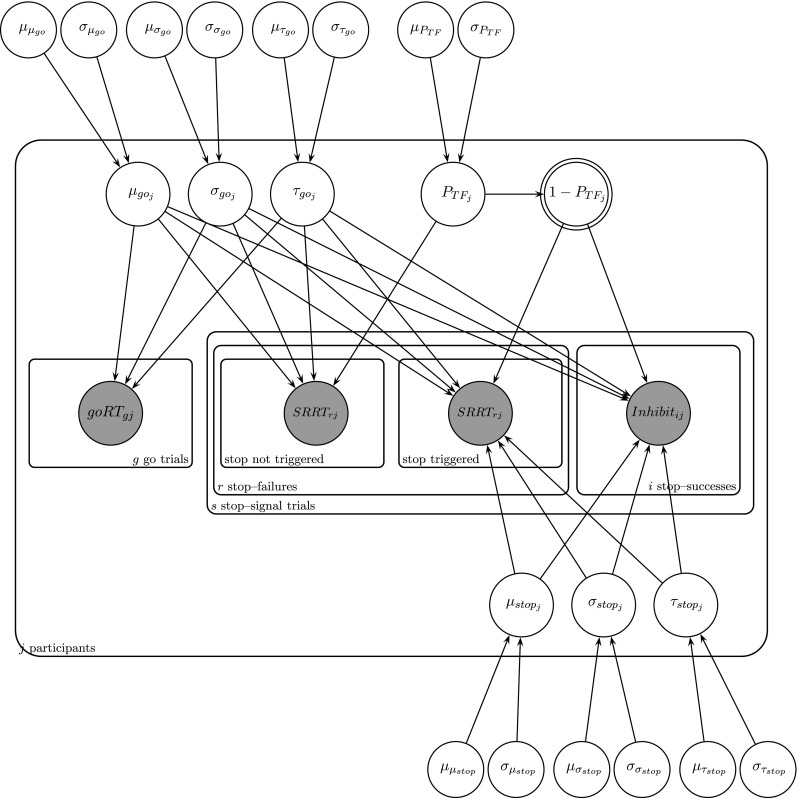
Directed acyclic graph of the trigger-failure approach. Observed variables (i.e., data) are represented by shaded nodes; unobserved variables (i.e., parameters) are represented by unshaded nodes. The graph structure indicates dependencies between the variables, and the plates represent independent replications of the participants (*j*) and the different types of trials (*g* for go trials; *r* for stop-failure trials, and *s* for stop-success trials). The participant-level go and stop parameters are modelled with truncated normal population distributions, with means and standard deviations estimated from data. The participant-level P_TF_ parameters are modelled on the real line after a probit transformation

A directed acyclic graphic representation (Lee, 2008) of the hierarchical trigger-failure model is shown in Fig. 2. Observed variables (i.e., data) are represented by shaded nodes; unobserved variables (i.e., parameters) are represented by unshaded nodes. The graph structure indicates dependencies between the variables, and the plates represent independent replications of the participants and the different types of trials.

The hierarchical model assumes that the go (μ_go_, σ_go,_ and τ_go_) and stop (μ_stop_, σ_stop,_ and τ_stop_) parameters for individual participants are drawn from truncated normal population distributions. Each participant’s trigger failure parameter ${P}_{TF}$ is modelled after a probit transformation by a truncated normal population distribution. The population distributions describe the between-subject variability of the parameters and are themselves characterized by a set of parameters—the population means and standard deviations—estimated from data. For instance, the participant-level μ_stop_ parameters are modelled with a truncated normal population distribution with mean μ_μstop_ and standard deviation σ_μstop_. Analysis of population-level parameters is appropriate for inference about a new sample of participants, analogous to a frequentist random-effects analysis. Priors used for the population-level parameters are weakly informative uniform and truncated standard-normal distributions (for details, see the Supplemental Materials on the Open Science Framework at https://osf.io/bxedk/).

In both data sets, the trigger-failure model was fit to the data of the two groups separately. Parameters were estimated using the BEESTS software (Matzke, Love et al., 2013). The resulting posterior distributions quantify knowledge about the parameters after the data have been observed; we used the median of the posterior distribution as point estimate for the parameters, and the 2.5^th^ and 97.5^th^ percentile of the distribution (i.e., 95% credible interval) to quantify estimation uncertainty. We used the Deviance Information Criterion (DIC; Spiegelhalter et al., 2002) to compare the descriptive accuracy of the model with and without the trigger-failure parameter.

## Results

For Badcock et al. (2002), a DIC difference of 117 for controls and 103 for schizophrenia patients indicated strong evidence for the model with trigger failures. The advantage for the trigger-failure model was even stronger for Hughes et al. (2012), with DIC differences of 236 and 237, respectively, for controls and patients. As shown in the Supplemental Materials, posterior predictive model checks (Gelman, Meng, & Stern, 1996) indicated that the trigger-failure model provided a good description of the go RT distributions and inhibition functions of most participants.

### Group differences

Table 1 presents the median and 95% credible interval of the posterior distributions of the population means of the go, stop and ${P}_{TF}$ parameters. Inference about group differences was based on overlap between the posterior distributions using Bayesian *p* values, the proportion of posterior samples that are lower in the schizophrenia group than in controls; *p* values close to 0 indicate that the posterior distribution of schizophrenia patients is shifted to higher values, and provide evidence for the presence of a group difference.^3^

**Table 1:** Medians and 95% credible intervals (CI) of the posterior distributions of population-level means of the go, stop and ${P}_{TF}$ parameters for Badcock et al. (2002) and Hughes et al. (2012)

		Schizophrenia		Control		Bayesian *p* value
		Posterior median	95% CI	Posterior median	95% CI	
Badcock et al. (2002)	μ_go_	444	[399, 492]	436	[374,494]	0.40
	σ_go_	66	[35, 80]	44	[4, 71]	0.13
	τ_go_	115	[14, 164]	70	[27, 87]	0.18
	Mean go RT	556	[449, 629]	503	[435, 565]	0.19
	μ_stop_	162	[128, 194]	144	[125,165]	0.18
	σ_stop_	26	[2, 50]	25	[3, 41]	0.48
	τ_stop_	20	[2, 52]	13	[2, 36]	0.35
	P_TF_	.17	[.07, .32]	.10	[.06, .16]	0.14
	Mean SSRT	185	[149, 214]	160	[137, 178]	0.10
Hughes et al. (2012)	μ_go_	434	[362, 500]	418	[377, 458]	0.34
	σ_go_	66	[25, 84]	54	[38, 65]	0.18
	τ_go_	85	[21, 112]	49	[26, 60]	0.09
	Mean go RT	516	[426, 592]	466	[422, 509]	0.14
	μ_stop_	180	[137, 213]	141	[130,151]	0.03
	σ_stop_	14	[2, 32]	9	[1, 18]	0.32
	τ_stop_	13	[2, 26]	12	[2, 19]	0.45
	P_TF_	.18	[.09, .31]	.07	[.04, .12]	0.02
	Mean SSRT	193	[150, 226]	153	[140, 162]	0.03

Population-level mean of the ${P}_{TF}$ parameters is transformed back to the probability scale; the inverse-probit transformed population-level ${P}_{TF}$ parameter approximates the *median* of the ${P}_{TF}$ parameters on the probability scale. The population-level *mean* of the ${P}_{TF}$ parameters on the probability scale can be computed by applying an inverse probit transformation that simultaneously considers the population-level mean and the population-level standard deviation. For the Badcock et al. (2002) data set, this transformation resulted in a posterior median of 0.24 for schizophrenia patients and 0.15 for controls, with a Bayesian *p* value of 0.06. For the Hughes et al. (2012) data set, this transformation resulted in a posterior median of 0.21 for schizophrenia patients and 0.09 for controls, with a Bayesian *p* value of 0.01.

For the go parameters, Bayesian *p* values did not indicate the presence of group differences in the μ_go_ parameter. In contrast, σ_go_ and τ_go_ were shifted to higher values for patients relative to controls in both studies, with Bayesian *p* values ranging between 0.18 and 0.09 in the two data sets. These results provide suggestive evidence that the slowing of mean go RT (μ_go_+τ_go_) in schizophrenia is largely attributable to slowing in the tail of the RT distribution (τ_go_), on average by 45 ms in Badcock et al. (2002) and 36 ms in Hughes et al. (2012).

For the stop and ${P}_{TF}$ parameters, Bayesian *p* values did not indicate the presence of group differences in σ_stop_ and τ_stop_. In contrast, μ_stop_ and ${P}_{TF}$ were shifted to higher values for patients relative to controls in both studies. For Badcock et al. (2002), there was suggestive evidence for the presence of a group difference in μ_stop_ and ${P}_{TF}$, with Bayesian *p* values of 0.18 and 0.14, respectively. For Hughes et al. (2012), there was strong evidence for a group difference in μ_stop_ and ${P}_{TF}$, with Bayesian *p* values of 0.03 and 0.02, respectively. The results indicated that group differences in stop performance are attributable to patients’ increased trigger failure probability and a slowing of mean SSRT as a result of a shift in the entire SSRT distribution due to an increase in μ_stop_.

### Exploratory analyses of ERP correlations

Research over the past 25 years has afforded a good understanding of the neural events underpinning the processing of the stop signal and the execution of the stop process. Work using event-related potentials (ERPs) has associated smaller N1 and P3 amplitudes to stop signals with stop failures and found that P3 peak latency to stop failures is delayed (De Jong et al., 1995; Bekker et al., 2005; Hughes et al., 2012). The reduced N1 and P3 amplitudes to stop signals during stop failures suggest that problems in early perceptual processing and lapses of attention could play a role in poorer stop-signal performance. To provide converging evidence for the trigger-failure account of stop-signal performance, we now report correlations between model parameters and Hughes et al.’s (2012) ERP data.

Our analysis examined the association between stop-related parameters and both N1 and P3 amplitudes and latencies at sites with maximal amplitudes (Cz and Fz, respectively). In particular, we focused on the stop-related parameters associated with group differences, ${P}_{TF}$ and μ_stop_ (results for mean SSRT were almost identical to those for μ_stop_). Inference about correlations used Bayesian “plausible values” (Ly, Boehm et al., in press; Marsman et al., 2016), avoiding overconfident effect size estimates associated with frequentist tests of hierarchical Bayesian estimates (Boehm et al., 2015). Our analysis also treated participants as random effect, thus imposing a very strict standard of evidence, taking into account uncertainty in generalizing from Hughes et al.’s (2012) small sample of participants to the population as well as posterior uncertainty about the participant-level parameters.

For each ERP-parameter combination, we computed sample correlations between the set of participant ERP measures and each participant-level posterior sample and then used Ly et al.’s (2016) analytical solution to compute the posterior distribution of the population correlation. The resulting population-level posteriors were averaged to arrive at a single posterior distribution for the population correlation. We used uniform prior distributions between −1 and 1 for the computation of the population-level posteriors. Sensitivity analyses indicated that the influence of the prior was negligible.

Inference was based on Bayesian *p* values for the proportion of samples in the posterior distribution of the population correlation above (for negative correlations) or below (for positive correlations) 0; *p* values close to 0 indicate that the posterior is reliably shifted away from 0. As shown in Table 2, properly taking into account all sources of uncertainty resulted in broad posterior distributions, with only the strong negative correlation between ${P}_{TF}$ and N1 latency in the schizophrenia group reliably differing from 0. This result indicates that higher levels of trigger failures were associated with an earlier N1 peak. Although individual variation in trigger failures was largest in patients (5-37%) it also was quite large in controls (2-18%), suggesting that differential range restriction was not the cause of the effect being restricted to the former group.

**Table 2:** Medians and 95% credible intervals (CI) of posterior distributions of population correlations between stop-related parameters and ERP indices for Hughes et al. (2012)

		Schizophrenia			Control		
		Posterior median	95% CI	Bayesian *p v*alue	Posterior median	95% CI	Bayesian *p v*alue
μ_stop_	N1 Cz amplitude	0.24	[−0.38, 0.72]	0.22	0.32	[−0.26, 0.73]	0.14
	N1 Cz latency	0.03	[−0.55, 0.59]	0.47	0.09	[−0.47, 0.59]	0.38
	P3 Fz amplitude	−0.29	[−0.74, 0.34]	0.18	0.11	[−0.44, 0.61]	0.35
	P3 Fz latency	0.06	[−0.52, 0.61]	0.42	−0.42	[−0.79, 0.17]	0.08
P_TF_	N1 Cz amplitude	0.31	[−0.32, 0.76]	0.16	0.02	[−0.53, 0.56]	0.48
	N1 Cz latency	−0.60	[−0.88, 0.01]	0.03	0.02	[−0.52, 0.56]	0.47
	P3 Fz amplitude	−0.41	[−0.80, 0.23]	0.10	−0.28	[−0.72, 0.30]	0.17
	P3 Fz latency	−0.25	[−0.72, 0.37]	0.21	0.13	[−0.44, 0.63]	0.33

## Discussion

In the data from both Badcock et al. (2002) and Hughes et al. (2012), trigger failures were more than twice as frequent in the schizophrenia than the control group, increasing from approximately 8.5% to almost 17.5%. If we had ignored trigger failures we would have substantially overestimated SSRTs. In the original papers, nonparametric SSRT estimates were slower in the schizophrenia than the control group by 31 ms for Badcock et al. and by 70 ms for Hughes et al. In contrast, our analysis that takes into account trigger failures produced reduced estimates of 25 ms and 40 ms, respectively. Thus, our results indicate that a substantial part of the reason that schizophrenia impairs the ability to inhibit motor responses is a failure of stop-cue processing, which leads to a failure to trigger motor inhibition mechanisms or to engage a brake to stop action (Aron et al., 2014).

Although allowing for trigger failures reduced the estimated slowing of the stop process, there was still evidence of residual slowing of SSRT in schizophrenia. However, our results suggest dissociation between the causes of slowing in the go and the stop process. In both data sets, for the go process, there was an increase in the proportion of slow responses in the tail of the distribution, due to an increase in τ_go_. For the stop process, in contrast, there was uniform slowing across the entire distribution, due to an increase in μ_stop_. It therefore is unlikely that the inhibitory disadvantage has the same underlying cause as the general slowing of choice responses in schizophrenia.

Based on the results of Matzke and Wagenmakers (2009), it seems likely that the increased τ_go_ is due to a decrease in the rate at which evidence about the choice response is accumulated (see also Heathcote et al. 2015). It is conceivable that the increase in τ_go_ also could reflect increased go threshold resulting from strategic slowing (Logan et al., 2014). However, as the two groups did not differ in μ_go_, it seems likely that the increase in τ_go_ purely reflects a decreased evidence accumulation rate in schizophrenia. In contrast, the increase in μ_stop_ is likely due to a deficit in the initial encoding of the stop signal and hence slowing of the initiation of the stop process, rather than a deficit in the rate at which the stop process runs. Once again, there is an alternative interpretation, that the increase in μ_stop_ reflects an increase in stop threshold. However, as the two groups did not differ in τ_stop_, the increase in μ_stop_ would be indicative of a higher stop threshold only in the unlikely scenario of a compensatory *increased* stop rate in schizophrenia. Taken together, therefore, our results are most consistent with attentional factors largely or completely mediating poorer ability to inhibit action in schizophrenia. The slowing in encoding processes supports the importance of elongated stimulus encoding in schizophrenia (Neufeld, 2007), which has been found to be more common in non-paranoid patients (Broga & Neufeld, 1981) and has been suggested to be due to additional constituent encoding operations (Taylor et al., 2016).

All of the patients in the studies that we analysed were medicated with one exception in Hughes et al. (2012). The majority were on atypical (or second generation) antipsychotics, which have not been associated with major cognitive impairments—rather the evidence suggests slight amelioration of deficits relative to first-generation antipsychotics (Hill et al., 2010). It therefore seems unlikely that any of the observed effects are due to side effects of medication.

Consistent with our behavioural results, ERP data from Hughes et al. (2012) suggest that patients are impaired in processing both visual go and auditory stop signals. For both modalities, the peak amplitudes of patients’ N1 and P3 components were smaller than for controls, and for auditory stop signals both N1 and P3 also peaked later in patients. Functional magnetic resonance imaging (fMRI) data revealed reduced blood-oxygen level dependent (BOLD) activation in left superior temporal gyrus in patients, consistent with reduced and delayed auditory ERP peaks, indicative of less attention to, and delayed sensory processing of, auditory stop stimuli.

We also found that more frequent trigger failures were correlated with earlier peaking auditory-evoked N1 components in patients but not controls. At first glance this finding might seem paradoxical, but it can be understood in the context of an attentional impairment in schizophrenia (McGhie & Chapman, 1961; Michie et al., 1990). It is well established that auditory-evoked N1 has multiple generators (at least six) modulating observed N1 amplitude and peak latency (Näätänen & Picton, 1987). Factors that influence these generators—and hence modulate auditory-evoked N1—encompass stimulus attributes, individual differences, and task factors, including attentional demands. Attention to auditory stimuli results in increased amplitude of N1 at 100 ms (Hillyard et al., 1973) and later negativities that overlap N1 and beyond up to 250 ms (Näätänen & Michie, 1979; Hansen & Hillyard, 1980). These later negativities are particularly reduced in schizophrenia patients (Michie et al., 1990; Ward et al., 1991). If a dysfunctional attention mechanism contributes to trigger failures in schizophrenia, we would expect this faulty mechanism to be associated with reduced auditory stop-signal N1 (as observed overall for patients by Hughes et al., 2012) and reductions in later attention-related negativities. Hence, our observation in schizophrenia patients of a negative relationship between trigger-failure propensity and earlier N1 peak latency may stem from the *degree* of dysfunction in the attention mechanisms reflected in reduced later negativities and therefore an apparently earlier peak latency of N1.

The fMRI data reported in Hughes et al. (2012) further support schizophrenia patients having a deficit in executing the inhibitory process, indicated by anomalous BOLD activation in their right inferior frontal gyrus (rIFG) during successful stop trials. Larger BOLD responses in rIFG for successful stop trials have been related to faster SSRT, and impaired function of rIFG has been linked to slower SSRT (Aron et al., 2014), leading to the argument that this structure is a key component of the stopping network. Hughes et al. found that underactivation of rIFG during stop responses accounted for patients’ slower SSRTs. However, there are competing theories regarding what aspect of stop-signal performance is reflected in rIFG activation, either the inhibitory process itself (Aron et al., 2006) or attentional processes involved in processing salient, task-relevant cues, such as stop signals (Hampshire et al., 2010). In the light of the current findings, and Hughes et al.’s neuroimaging data, we propose longer SSRTs and enhanced trigger failures in schizophrenia derive from dysfunction of the attentional role rIFG has in encoding stop signals.

Overall, our results indicate patients’ poorer stop-signal performance is not due to a deficit in the inhibitory process itself, but rather is due to sensory or attentional deficits associated with stop-signal processing. Hearing loss is a risk factor for psychosis (Linszen et al., 2016), but it is unlikely that patients failed to hear Badcock et al.’s (2002) and Hughes et al.’s (2012) auditory stop signals as they were highly salient (e.g., ~85 dB SPL in Hughes et al.), so it seems more likely that the locus of the deficit is more central. Alternatively, patients could have had difficulty switching their attention between the visual go signal and the auditory stop signal. However, SSRT deficits are found in schizophrenia patients even when both go and stop signals are visual (Enticott et al., 2008; Thakkar et al., 2011, 2015). It also is possible that the problem resides in processes initiating inhibitory processing in response to the detection of a stop signal.

More generally, our results indicate that attentional factors should be considered when interpreting performance in the stop-signal paradigm. We found that trigger failures occurred on a substantial proportion of trials in control participants, and that trigger-failure rate was doubled in a clinical population known to have a dysfunction of attention (Braff, 1993). Therefore, we advise that the possibility of trigger failures and slowing in the initiation of inhibitory processes be assessed in any application of the stop-signal paradigm, even with participants who do not have any known attentional deficits. Future research might also examine whether elevated levels of these factors are present in other disorders of attention, such as attention-deficit hyperactivity disorder, which is commonly found to be co-morbid with schizophrenia (Levy et al., 2015). Further validation of the attentional account of stop-signal performance would be gained if trigger failures were found to vary with factors known to affect attention, such as fatigue, and if the somewhat surprising changes we observed in attention-associated ERP waveforms were replicated in a larger sample.

## Footnotes

1 A reviewer pointed out that attentional affects could also manifest in τ_stop_ under different assumptions to those made by Matzke and Wagenmakers (2009), such as a multi-step encoding process, where on some trials only one or two of the steps are affected by attention deficits associated with schizophrenia.

2 From the original Badcock et al. (2002) sample, data from 3 patients and 3 controls could not be retrieved.

3 The frequentist decision rule to reject the null hypothesis if *p* < 0.05 does not apply to Bayesian *p* values, which quantify the degree to which the posterior distribution of the difference is consistent with the hypothesis that the parameter is greater for schizophrenia patients than for controls. For instance, a Bayesian *p* value of 0.10 indicates that the hypothesis holds in 90% of the posterior distribution of the difference.
